# Cavity Ring-Down Methane Sensor for Small Unmanned Aerial Systems

**DOI:** 10.3390/s20020454

**Published:** 2020-01-14

**Authors:** Benjamin Martinez, Thomas W. Miller, Azer P. Yalin

**Affiliations:** 1 Department of Mechanical Engineering, Colorado State University, Fort Collins, CO 80525, USA; Benjamin.Martinez2@colostate.edu; 2 TCB Engineers, Surprise, AZ 85374, USA; tmiller@tcbengineers.com

**Keywords:** methane, natural gas, oil and gas, landfill, cavity ring-down spectroscopy, spectroscopy, laser absorption, small unmanned aerial system, unmanned aerial vehicle, drone

## Abstract

We present the development, integration, and testing of an open-path cavity ring-down spectroscopy (CRDS) methane sensor for deployment on small unmanned aerial systems (sUAS). The open-path configuration used here (without pump or flow-cell) enables a low mass (4 kg) and low power (12 W) instrument that can be readily integrated to sUAS, defined here as having all-up mass of <25 kg. The instrument uses a compact telecom style laser at 1651 nm (near-infrared) and a linear 2-mirror high-finesse cavity. We show test results of flying the sensor on a DJI Matrice 600 hexacopter sUAS. The high sensitivity of the CRDS method allows sensitive methane detection with a precision of ~10–30 ppb demonstrated for actual flight conditions. A controlled release setup, where known mass flows are delivered, was used to simulate point-source methane emissions. Examples of methane plume detection from flight tests suggest that isolated plumes from sources with a mass flow as low as ~0.005 g/s can be detected. The sUAS sensor should have utility for emissions monitoring and quantification from natural gas infrastructure. To the best of our knowledge, it is also the first CRDS sensor directly deployed onboard an sUAS.

## 1. Introduction

Detection and quantification of methane (CH_4_) emissions is a topic of increasing importance given methane’s role as a greenhouse gas and its increasing atmospheric concentration [1]. The Intergovernmental Panel on Climate Change (IPCC) reports a 20-year Global Warming Potential (GWP_20_) for methane that is 84 times that of CO_2_ [2]. The IPCC also reported that, in 2010, CH_4_ accounted for 16% of the total anthropogenic GHG emissions based on GWP_20_. Methane is also a precursor to tropospheric ozone formation [3], which itself has negative environmental and health consequences, through photochemical reactions of methane with nitrogen oxides [4]. Major anthropogenic sources of methane include agriculture [5] and landfills [6], as well as activities associated with natural gas production, distribution, and end-use [7]. (Note that natural gas typically contains ~80–95%, or higher, methane by mass fraction).

Our focus herein is largely in connection with emissions monitoring for natural gas infrastructure, but similar sensor approaches can be used for other localized sources. New drilling techniques, in particular hydraulic fracturing (commonly referred to as fracking), have led to a dramatic rise of gas production around the world, particularly in the United States. In our geographic region of the state of Colorado there is estimated to be more than 40,000 wells, with more than 10,000 new wells created since 2005 [8]. As a first step towards mitigating the associated methane emissions it is critical to have suitable monitoring techniques. Given this backdrop, there has been an intensive effort from academia and industry to develop appropriate new sensing technologies [9,10]. 

A full review of all methane sensing approaches is beyond the scope of the present contribution and we limit our focus to sensor deployment on small-unmanned aerial systems (sUAS), alternately referred to as Unmanned Aerial Vehicles (UAVs), and sometimes drones. We define an sUAS as a flight platform, rotor-based or fixed wing, with an “all-up” mass of 55 pounds (25 kg) or less, such that in the United States it falls under the Small Unmanned Aircraft Regulations (Part 107) per the Federal Aviation Administration. The recent proliferation of these sUAS platforms is enabling for many new applications related to environmental sensing [11]. Several groups (e.g., [12]) have examined the use of very compact (<~1 L) and inexpensive methane sensors, based on non-dispersive infrared, but found them to have inadequate sensitivity (>~1 ppm) and in some cases to suffer from interferences. A different approach [13] consists of gathering a series of air samples during flight, into a tube filled by a pump, for subsequent analysis on the ground. In general, however, real-time aerial measurements are more convenient. High-end analyzers of larger mass (>~15 kg), as are typically used for sensitive ground-based measurements, can be deployed on larger UAS (as opposed to sUAS), such as the NASA SIERRA aircraft (mass ~250 kg) [14]. The same class of sensors can be deployed via sUAS by keeping the sensor on the ground and flying only the inlet, which passes sample air to the sensor through a tube (~100 m length), but such arrangements are logistically more complex and can introduce artifacts associated with the flow lag-time through the rather long tube [15,16]. 

Other recent sensors combine low mass (<~5 kg), low power (<~30 W), and high sensitivity (<~0.1 ppm), making them better suited to sUAS deployment [17,18,19,20,21,22]. Zondlo and collaborators have demonstrated compact laser sensors (meeting these specifications) based on wavelength modulation spectroscopy (WMS) with Herriot cells for use on both fixed-wing and hexacopter sUAS [17,18]. Researchers at the Jet Propulsion Laboratory (and collaborators) report a related sensor, also demonstrated on a hexacopter sUAS, where the sensor was mounted on a boom to reduce influence of rotor downwash [19]. Another group reports a compact laser sensor integrated to a fixed wing sUAS though noise in the flight-data called for detection thresholds in the 0.3–1 ppm range [20,21]. The sensor we present herein uses a different spectroscopy technique but is most similar, in broad attributes, to this class of (point-measurement) laser absorption sensors (i.e., [17,18,19,20,21,22]). Similar laser techniques can be applied, also in compact packages, in backscatter configurations where the beam is directed out of the sUAS sensor to a hard target from which scattered light is collected (with a detector on the sensor). In this case, rather than providing a point concentration, the sensor measures the path-integrated concentration along the beam (sensor to target), which is typically pointed downward from the sUAS to the ground [23,24]. Yang et al. have reported a detection limit of ~5 ppm-m, which corresponds to (average) concentrations of ~5–0.5 ppm for nearfield plumes of thickness ~1–10 m. The authors report challenges in determining the methane background for flights at a height above 10 m [24].

Here, we report on a purpose built sensor for mobile methane sampling from sUAS. The sensor is based on the cavity ring-down spectroscopy (CRDS) technique allowing sensitive (and specific) methane detection. We use an open-path configuration where air that flows through the open-cavity (mounted just below the sUAS), allows for a high temporal response (1 s), low-power (12 W), and high sensitivity (<~30 ppb) in a compact design amenable to sUAS integration. 

## 2. Methods

### 2.1. Open-Path Cavity Ring-Down Sensor

**Figure 1:**
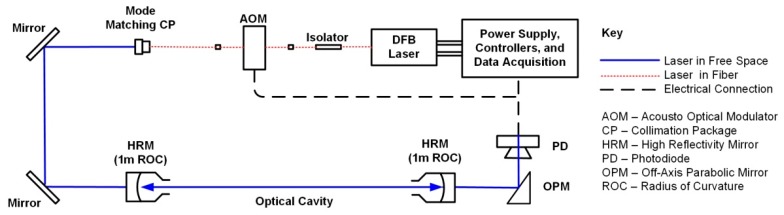
Schematic diagram of CRDS methane sensor.

The methane sensor presented here employs the laser absorption cavity ring-down spectroscopy (CRDS) technique where the sample (ambient air in our case) is measured within a high-finesse cavity to achieve high detection sensitivity [25,26]. To the best of our knowledge, this is the first report of a CRDS sensor (for any species) deployed onboard an sUAS. Past publications from our group provide further details on the spectroscopic scheme and laboratory testing of the sensor [27,28] with essential details provided here. Figure 1 shows a schematic of the sensor. The laser source is a continuous-wave distributed feedback (DFB) diode laser (NEL/NTT) in a 14-pin butterfly package operating at ~1651 nm (see below). To record spectra, the laser is current-scanned with a compact controller (scan details below). A thermo-electric cooler (TEC) fixes the laser temperature to locate the scan region. An inline fiber isolator prevents back reflections from the cavity to the laser. Light exciting the final fiber after the AOM is approximately 5 mW in power and is passed to free space through an adjustable focus aspheric FC collimator (Thorlabs CFC-8X-C, Newton, NJ, USA). This collimator is adjusted to achieve spatial mode matching to the optical cavity [29], which is comprised of two high-reflectivity (HR) mirrors separated by 60 cm. The HR mirrors have dielectric coatings on fused silica substrates and have reflectivity of R ~0.99994 at 1651 nm, corresponding to an optical path length of ~10 km. Light exiting the cavity is measured with a high-gain low-noise InGaAs photodetector amplifier module (Analog Modules, 712B-4-DC). We employ a trigger system that looks at light transmission through the cavity to monitor for light injection to the cavity, at which time light to the cavity is rapidly extinguished with an acousto-optic modulator (AOM) to produce ring-down decay events [30]. 

The overall sensor logic and control is handled by a National Instruments sbRIO-9651 based on the Xilinx Zynq-7020 system on chip (SoC) coupled with a custom carrier board to form the electronics platform core. Acquisition, triggering, and laser scanning are implemented on the FPGA side of the SoC and ring-down fitting and analysis reside on the dual-core ARM Cortex-A9 CPU running a real-time operating system. Details of the laser scanning and spectral fitting are summarized as follows. The laser is continuously scanned (1–10 Hz) across a range of interest (~1650.89–1651.03 nm) containing a methane absorption feature. When the trigger circuit (summarized above) fires, the detector records a ring-down decay signal that is fit with a linearized exponential by an iterative nonlinear least squares method. The ring-down ingestion rate varies according to threshold trigger values but is generally in the range of ~350–500 Hz. Ring-down times are then converted to absorption coefficients, and associated with their laser frequency, to yield the absorption spectrum. Details of the lineshapes and spectral fitting equations are provided in our past references [27,28]. In summary, in this region there are 4 methane absorption lines that, at atmospheric pressure, show up as one effective peak. The measured spectrum is then fitted with a synthetic spectrum with 3 free fit-parameters: the methane concentration, the center frequency position, and the baseline loss (which corresponds to the mirror reflectivity). The fit uses parameters from the HITRAN 2012 database [31]. Fitting requires knowledge of temperature and pressure which are recorded with standard compact sensors (Omega RTD-806 and Honeywell PX2AM1XX001BAAAX). (These sensors were deployed on the ground for the work presented herein, but we have also integrated them within the electronics enclosure and shown aerial data logging.) Finally, a GPS sensor (Linx, RXM-GPS-FM-T) within the electronics enclosure records data for time-stamping and spatial localization.

### 2.2. Hexacopter sUAS and Sensor Integration

The selection of suitable sUAS platforms for air quality sensing has been discussed in the literature (e.g., [11] and references therein). For ease of deployment on a versatile sUAS platform, we have employed a rotor-style hexacopter (DJI Matrice 600). The selected hexacopter is capable of vertical take-off and landing (VTOL) without need of a dedicated runway. As compared to fixed-wing aircraft, the VTOL hexacopter offers more flexibility in being able to fly near ground level with variable flight speeds, including the ability to be able to hover (loiter). With waypoint navigation, or manual control, it is possible to perform a series of transects of methane plumes downwind of emission sources (e.g., raster, staircase, spiral patterns, etc.). Flight operations, including all aspects related to safety and regulatory compliance were supported by the Colorado State University Drone Center.

**Figure 2:**
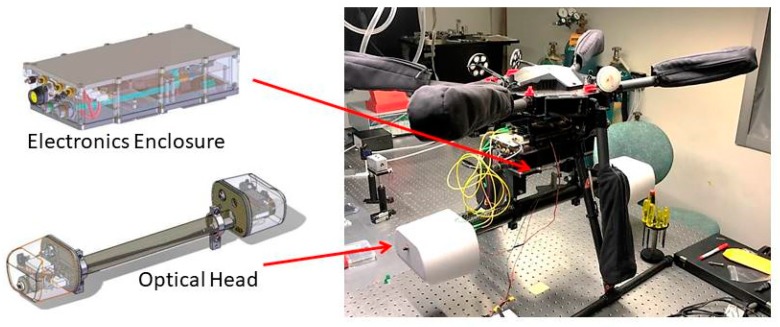
Illustration of sensor mounting to sUAS.

The maximum load capacity of the Matrice 600 is 6 kg, but to keep flight times to a maximum, the payload weight was minimized to ~4.1 kg. The payload consisted of two main components: the optical head (i.e., the high-finesse cavity through which sample air is measured) and the electronics enclosure (i.e., the module containing the micro-controller, circuit boards etc.). The head has approximate dimensions of 84 cm × 18 cm × 15 cm, with a mass of 2.0 kg, while the electronics enclosure has approximate dimensions of 29 cm × 14 cm × 8 cm, with a mass of 1.4 kg. The remaining portion (0.7 kg) of the 4.1 kg mass is due to the mounting hardware. The modules, and their mounting to the sUAS, are shown in Figure 2. (In actual flights the yellow fiber optic cables were coiled and secured to the sUAS body.)

The configuration used for the sUAS and shown in Figure 2 is substantially smaller and lighter relative to what we have deployed on automobiles [28]. In the case of automobiles, where we did not optimize for size and weight, and also had a gateway for cellular communications, the electronics box had dimensions of ~40 cm × 40 cm × 15 cm and mass of ~22 kg (excluding roof-rack mount). The smaller enclosure for the sUAS is made possible by forgoing the cellular components, tighter layout and packing of the custom circuit boards, and tighter packing of all hardware in a smaller enclosing box (from TIVAR 1000 EC plastic). Further, the head used for the sUAS is similar in design to that used on the automobile, both with a cavity length of 60 cm and similar optical components, but the sUAS version is ~40% lower mass due to slightly different shapes (and thinning) of the carbon fiber head structure and removal of the heater elements (see below).

The electronics enclosure was attached to the undercarriage of the Matrice 600 with gimbal mounting connectors (Z15 series) which attach to the enclosure with a 3-D printed plastic plate. Two mounting brackets machined out of ABS plastic attach the optical head with the top of the head ~31 cm below the undercarriage of the sUAS. With the legs of the sUAS deployed, the bottom of the head is ~14 cm above the ground (Figure 2). All wires connecting between components were shortened as much as possible to reduce mass. (As a temporary step, owing to a design change from a free-space to fiber-coupled AOM, in the flight reported here the AOM was affixed to a thin (<1 cm) mounting plate affixed to the outside of the electronics enclosure.) The sensor has power draws of ~12 Watts and can be powered with its own batteries or via the batters system of the sUAS. For the flights reported here, we have powered the sensor with an 18 V connection from the sUAS battery (DJI TB47S). The sUAS, with sensor mounted, has typical specifications as shown in Table 1.

### 2.3. Controlled Release Setup

**Figure 3:**
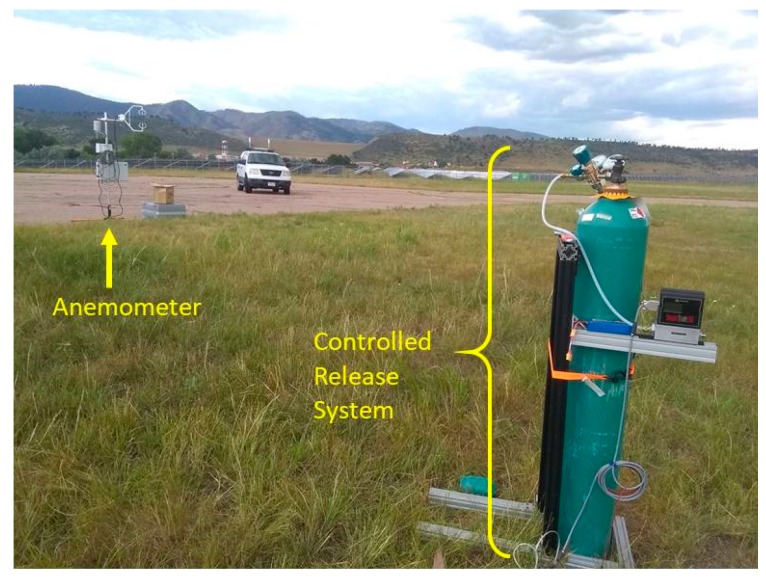
Photograph of controlled release setup.

Demonstrative sUAS flights were performed at Christman Airfield located in Fort Collins, Colorado. To experiment with plume detection by the sUAS methane sensor, we employed a controlled release setup to provide methane plumes of known mass flow. The setup included a cylinder of chemically pure (99.5%) methane delivered with a mass flow controller (Sierra SmartTrak 50). The final release point (mass flow controller outlet) was at a height of 1 meter above the ground. A weather station (Campbell Scientific, CR1000 and NL115) with ultrasonic anemometer (Campbell Scientific, CSAT3) was used to simultaneously record time series of wind speed amplitude (20 Hz rate). Plumes could be captured at varying distances up to ~500 meters from the source. Flight paths around the emission point were adjusted depending on wind magnitude and direction. The controlled release setup is shown in Figure 3.

## 3. Results and Discussion

### 3.1. Flight Performance of sUAS

**Figure 4:**
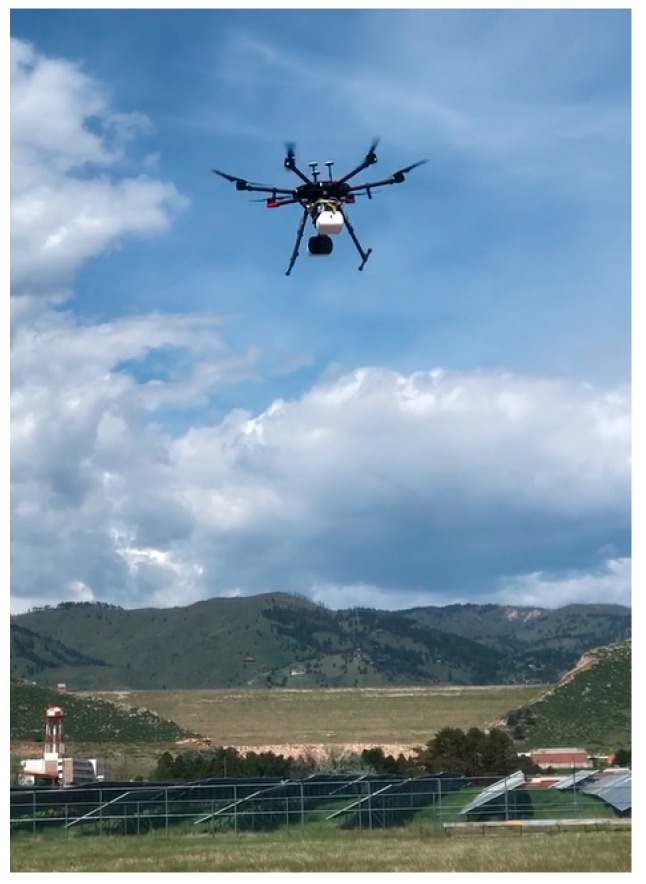
Photograph of sUAS in the air with integrated methane sensor.

Multiple successful flights were performed with the sUAS integrated methane sensor. A total of 22 controlled releases were performed at Christman Airfield (Fort Collins, CO, USA) in flights over several months during 2019. The mounting of the sensor did not noticeably affect the aerodynamics or ability to control and navigate the aircraft. Figure 4 shows a photograph of the sUAS sensor in the air.

### 3.2. Methane Detection Sensitivity

**Figure 5:**
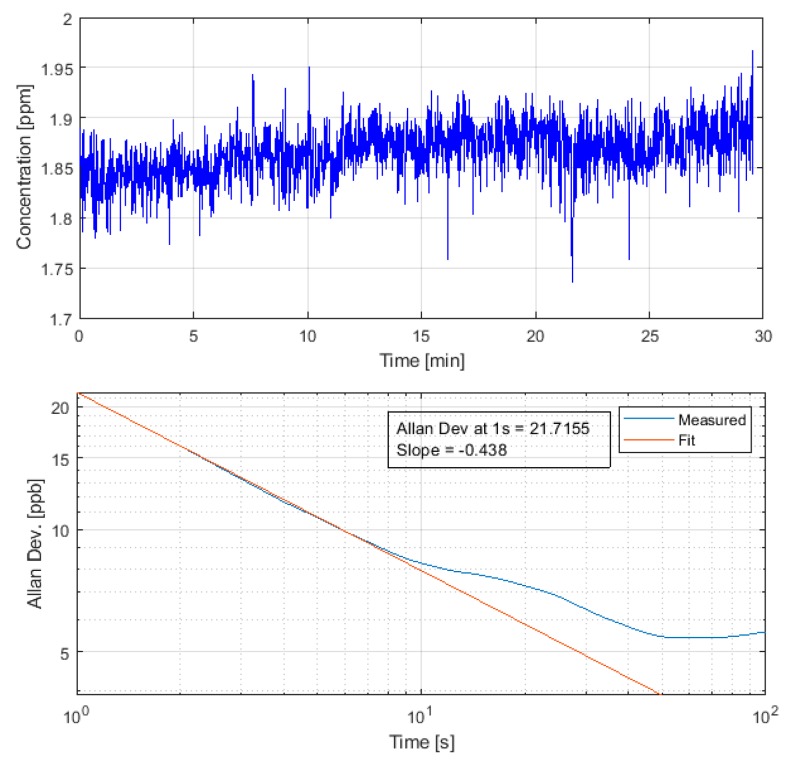
Allan deviation analyis of methane flight data from sUAS.

We have analyzed methane time-series obtained from the flying sensor to examine methane detection sensitivity. Specifically, we analyze the Allan Deviation [32,33] with example (typical) results shown in Figure 5. These measurements were performed without known methane sources in the vicinity but may be weakly influenced by any true variations in methane concentration. Looking at several patches of similar data, we find Allan deviations in the range of ~15–30 ppb (referenced to 1-s collection time). Similar tests in the laboratory, using a closed path with fixed concentration of ~2 ppm, yielded Allan deviations of ~10–5 ppb (for 1-s). The slight degradation in the flight value is attributed primarily to the effect of airbone dust particles (Mie scattering), perhaps with a secondary effect of vibration and thermal induced degradation of cavity alignment. Nonetheless, the senstivity (Allan deviation) of the flying sUAS configuration is very comparable to that of the driving (automobile) configuration of the sensor [28]. We conclude that the sensor maintains its sensitivity during sUAS flight and that vibrations or other effects do not cause noticeable performance degradation relative to ground-based mobile (automobile) deployment. Note that for automobile deployment we have developed a heater system for the sensor to counter the effects of ambient temperature variation on sensor alignment. For mass reasons, we do not currently use the heaters in the sUAS deployment. The flights performed to date have spanned a temperature range of ~10 K, and future work will further examine sensor stability over temperature. Maintaining high-sensitivity also requires that the HR mirrors maintain their reflectivity, and it was found that over the course of more than 10 flights, there was no noticable drop in reflectivity, nor a need to realign the sensor.

### 3.3. Methane Plume Detection

**Figure 6:**
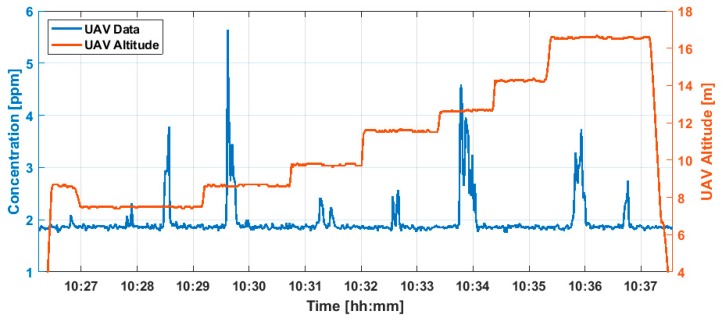
Plume detection by sUAS sensor due to controlled release of mass flow 0.5 g/s.

We have performed a series of controlled release tests to demonstrate the ability of the sensor to detect methane plumes representative of those produced by oil and gas infrastructure. Figure 6 shows an example methane time-series, along with altitude, for a flight of ~11 minutes duration. In this case the sUAS flew transects through the methane release plume at multiple altitudes between ~5 and 20 m at downwind distances in the range of ~75–150 m. The methane release had a mass flow of 0.5 g/s and the wind speed was ~1.5 m/s on average (and ~4 m/s maximum gust). Several plumes are clearly visible as the sUAS flies through regions of elevated methane concentration. The accuracy of the spectroscopic scheme in terms of plume amplitude has been examined in past closed-path laboratory tests which showed that measured concentrations agreed with expected values within experimental uncertainty (~15%) [28].

**Figure 7:**
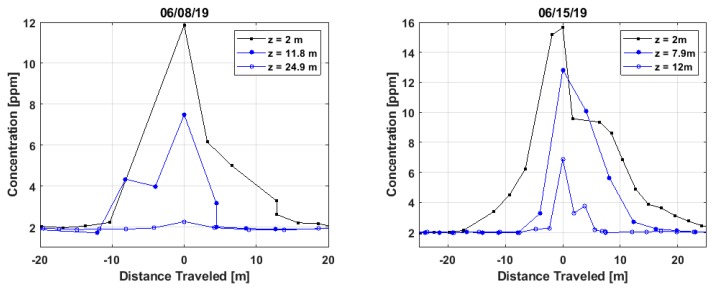
Examples of plume transects as the sUAS flies at different altitudes.

Figure 7 shows example plume transects at different heights for controlled releases of 0.5 g/s methane. The individual transects are at varying downwind distances from the source, with the sUAS flying in a straight line direction approximately perpendicular to the wind. The horizontal axis, Distance Traveled, is determined from the spacing of the sequential latitude/longitude readings. The transects in the left panel (8 June 2019) at an average distance of ~60 m from the source and those in the right panel (15 June 2019) at an average distance of ~70 m. On both days, wind speeds were ~2 m/s (average). Note that the transects at altitude 2 m were taken with an automobile mounted sensor. Turbulence causes the structure of the plume to continuously vary and different plume shapes are visible over the short time-scale (~10 s) of the transects. The data at 24.9 m altitude, in the left panel of Figure 6, exhibits only a very weak peak and is indicative that the sUAS flight-path was close to the top edge of the plume. The example plumes in Figure 7 have peak methane concentrations (above the ambient background) in excess of ~10 ppm. If we adopt a minimum threshold for plume detection of 100 ppb relative to the background (based on sensitivity of ~15-30 ppb), then, for the conditions of these flights (i.e., wind speed and downwind distance), it would be possible to detect isolated plumes with mass flows ~100 times lower than used here, so mass flows as low as ~0.005 g/s. Plume detection at this mass flow value should be experimentally confirmed, but this result is consistent with measurements by our similar automobile mounted sensor where we have detected plumes due to 0.03 g/s (at ~60 m) with quite high signal-to-noise [27].

**Figure 8:**
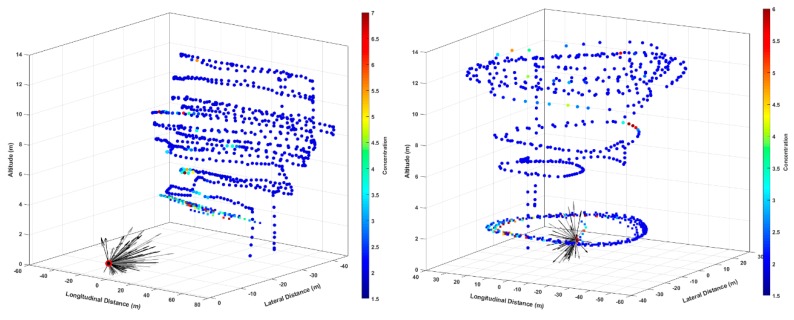
Geo-located methane concentration data from sUAS sensor from two flights. In both panels, the emission point is shown as a red circle (at altitude zero) with a time-series of wind vectors.

The 3-dimensional structure of the plume and possible flight paths are also illustrated in Figure 8 for controlled releases with a mass flow of 0.5 g/s. The left panel shows a raster type scan at an approximately fixed downwind distance, while the right panel shows a flight path comprised of approximately circular loops around the emission point at different heights. In both panels, the location of the emission point is shown as a red circle (at altitude zero) with a time-series of wind vectors also shown. (The wind-vectors were averaged from the raw 20 Hz readings to 1 Hz, and only 1 in every 15 of the 1 Hz readings is plotted for visual clarity). As discussed in our conclusion, by flying down-wind or around, the source one case use control-volume methods to infer the methane source mass-flow from the methane concentration fields and wind data.

## 4. Conclusions

The confluence of increased concern over methane emissions with increased use of sUAS platforms leads to a need for sensitive, compact, low-power sensors for methane measurement. As discussed in the introduction, there is currently a fairly limited set of appropriate sensor options, in particular with adequate part-per-billion level sensitivity, and the open-path CRDS methane sensor presents a candidate sensor approaching this goal. As a sensor that measures point concentration (as opposed to path integrated), and with sensitivity in the ~10–30 ppb range for 1-second response time, it is most similar in broad features to WMS sensors developed for similar purposes (e.g., [17,21,34]). Tradeoffs between CRDS and WMS sensors, in particular for practical field use, should be further investigated in the future. For example, the on-axis CRDS beam, versus use of larger mirror diameter optical cells in WMS, may mean that CRDS systems can be smaller or more reliably maintained. On the other hand, in CRDS, one must maintain very high mirror reflectivity, and one may suffer more from effects of ambient temperature variation on sensor alignment (given the more precise alignment requirements of high-finesse cavities). The role of these issues in long-term field use of the sUAS sensor will be further investigated in future work.

In regards to the choice of sUAS flight platform, and optimal integration of sensor to the platform, the effects of the flow field of the sUAS should be further considered. In particular, the effect of the downwash caused by the rotating rotor blades [35,36] can distort the shape (concentration field) of the methane plume, which could then complicate the use of algorithms to infer source mass flow based on the measured flow field. Indeed, future research will focus on inferring mass-flow (and source location) from the sUAS methane concentration maps and wind data [15,37,38,39].

## Figures and Tables

**Table 1:** Flight specifications of sUAS with methane sensor.

sUAS Platform	DJI Matrice 600
Mass	13.2 kg
Battery	TB47S
Max. Duration	~12 min
Typical Speed	2.7 m/s
